# Theoretical and Experimental Analysis of Inter-Layer Stresses in Filament-Wound Cylindrical Composite Structures

**DOI:** 10.3390/ma14227037

**Published:** 2021-11-20

**Authors:** Piotr Krysiak, Aleksander Błachut, Jerzy Kaleta

**Affiliations:** 1 Military Institute of Engineer Technology, 136 Obornicka Street, 50-961 Wroclaw, Poland; 2 Department of Mechanics, Materials and Biomedical Engineering, Faculty of Mechanical Engineering, Wroclaw University of Technology, 27 Wybrzeże Wyspiańskiego Street, 50-370 Wroclaw, Poland; aleksander.blachut@pwr.edu.pl (A.B.); jerzy.kaleta@pwr.edu.pl (J.K.)

**Keywords:** composite rings, analytical modelling, numerical simulation, interlayer pressure, residual stress

## Abstract

This paper analyses the issues relative to the modelling of tubular (cylindrical) composite structures. This paper aims to describe the design of a multi-layer structure of filament-wound composite pipes where, after loading, the hoop-stress distribution would be as uniform as possible. That would allow the mass of the composite to decrease while maintaining the proper mechanical strength. This publication presents the development of a calculation model dedicated to mono- and multi-layered tubular composite structures. The equations describing the stress pattern were based on the Lamé Problem, whereas to describe the modelled structures, an anisotropy coefficient was introduced and interlayer pressures values were determined. To verify the calculations, experimental studies were performed. The test specimens were fabricated by winding fibre bundles around a steel core (as rings with an internal diameter of 113 mm and a height of 30 mm). For the test, the method of pressing a conical ring into a split ring, which acts on the internal surface of the tested cylindrical sample, was selected. The operation of the test rig (test stand) was simulated using the Finite Element Method (FEM). Measurements with strain gauges were conducted during the experiments.

## 1. Introduction

The developments in the area of using reinforced polymer composites for the production of lightweight structures, enabling the transfer of high loads, has resulted in the development of newer and newer reinforcing fibres characterized by high strength.

Typical construction materials are generally homogeneous and, in most cases, isotropic in nature. Composites, on the other hand, are heterogeneous materials, the vast majority of which are also anisotropic (or orthotropic, with deliberate reinforcement arrangement). This means that the properties of these materials, including the mechanical properties, are a function of the location of the tested volume element and the direction of the load acting on it. Therefore, as far as strength calculations are concerned, the use of composites in engineering applications requires the use of dependencies other than those observed for traditional, homogeneous construction materials, both relative to elasticity, and in limit regions determined by the strength of the material.

The object analysed in this study is a pipe composed of a continuous fibre reinforcement phase embedded in a continuous polymer matrix. From the point of view of the technology involved, such a pipe is an axisymmetric object in which the parameters of the filament winding (winding angle, winding tension) can be easily tailored. From the modelling point of view, it is an axisymmetric object with an anisotropic (orthotropic) structure, either single- or multi-layered, with the same or different structure of elementary layers, and with possible additional boundary conditions between the layers. What follows is that even for geometrically uncomplicated composite structures, the analytical description should include complex problems relative to the material strength. Several works dedicated to the analysed subject matter are presented below.

One study [1] considered the problem of the elastic equilibrium of a homogeneous anisotropic body with a cylindrical cross-section, loaded with external forces which cause a state of stress only along two coordinates (stress does not change along the length). Another study [2] included the introduction of basic equations that were derived for an elastic body characterized by cylindrical anisotropy in a plane stress state and a plane strain state, while in [3], equations for the distribution of radial, circumferential- and longitudinal stresses were determined for a pipe made of anisotropic material (oakwood). In [4], the author determined the equations describing the state of stress of various structures, from plates and shells to curved bars and pipes, considering anisotropy and various load cases. Particularly noteworthy is the study on the description of the stress of an axisymmetric element, which consists of any number of layers in the form of concentric rings of equal thickness and the characteristics of anisotropic material, pressure-loaded on the inner- and outer surfaces.

The works cited provided a mainly theoretical basis for the various materials used at that time, as composite materials were not widely known and were not used on such a scale as they are today. In recent years, with the development of numerical methods and computer processing capabilities, as well as advances in materials (reinforcing fibres, resins) and manufacturing technologies, attempts have been made to model and identify the stress state of more complex composite structures.

In [5], the authors investigated elastic-plastic displacement fields in a thick-walled, unidirectionally reinforced pipe loaded with a uniformly distributed internal pressure. Following the analysis, formulas for ultimate tensile strength and ultimate working pressure were obtained. The authors of [6] presented a solution to the problem of stress distribution in a thick-walled composite pipe subjected to axial- and radial loading. The load ratio was determined with the appropriate proportionality factor. As a result of the theoretical analysis, the stress state of the structure under such loads was determined and it was found that the stress components in the pipe depend on the tube geometry, the proportionality factor, the hardening exponent, and the internal pressure value. Based on the knowledge of the proportionality factor and the hardening exponent, it can be stated whether the failure of the pipe would occur at the inner surface of the tube or the outer one.

Hamed et al. [7] aimed to determine the strain of the inner and outer surfaces of a thick-walled filament-wound composite pipe. The solid thick-walled cylinder theory was used for the calculations. The cylinder had been manufactured of laminated material with linear elastic properties. Various states of stress and strain at the outer and inner surfaces of the tube and the distribution of radial stress were considered. A computer program was written in FORTRAN language, which allowed the determination of stresses and strains in cylinders made of orthotropic materials. This program was developed based on the algorithm of a three-dimensional radial stress analysis, and the calculations were made on the example of a glass fibre-reinforced pipe. A review of classical formulas based on the theory of elasticity, used for structural analysis and determination of hoop stress in cylindrical and spherical pressure vessels was included in [8]. However, such formulae are mainly used for thin-walled structures. This paper presented applicability ranges for these dependencies. Another study [9] presented the exact three-dimensional thermoelastic solution for a composite pipe using the heat transfer equation along the thickness, by taking into account the body forces created by rotation and the Coriolis effect. The nonlinear dynamics of a simply-supported fluid-conveying composite pipe subjected to axial tension in sub- and super-critical regimes were investigated in [10], while the study [11] included a new constitutive model being developed to predict the elastic behaviour of plain weave textile composites, using the finite element (FE) method. The basics of a continuum theory developed for the plane curved composites were used for determining the geometric conditions and basic assumptions of the model. In [12], a particular failure criterion is proposed to predict failure in the composite structures with a reasonable margin of safety. The enhanced model was implemented into the commercial finite element software of ABAQUS via a developed user material (UMAT) subroutine, which utilized a suitable solution algorithm. A displacement model for micro and nano laminated composite Timoshenko beam was presented in [13]. The layer-wise displacement model was refined to calculate interlaminar stresses correctly and the behaviour of displacements at the interface of adjacent layers was modelled by transverse and tangential springs of high rigidity.

There is also a full range of studies dedicated to the effect of the wind angle during the winding process on the strength of the structure itself. The main focus hereof is on the circumferential winding with a wind angle of 90°. Nevertheless, the articles [14,15,16,17,18] deserve due attention.

The above reports are mainly theoretical considerations. Due to the complex nature of the composite structure itself, experimental verification is essential. Therefore, some examples of research work on the analysed issue are also discussed herein.

As part of [19], three barrels of 120-mm gun tubes were fabricated and tested experimentally. The barrel structure consisted of three layers: a steel liner, an insulating layer made of glass fibre–reinforced epoxy resin and an epoxy-carbon fibre support layer. Numerical analysis and experimental results showed the legitimacy of applying the above-mentioned approach to the design of this type of structure. Another study [20] presented the experimental results of quasi-static and impact indentation testing performed on filament wound glass fibre-reinforced epoxy resin pipes used in underwater systems. The study showed that the low impact energy causes a large decrease in implosion pressure resistance, whereas the damage caused by static loads is similar to impact damage, however its scale is much smaller.

A model describing the winding process in the case of a thick-walled composite pipe was developed in [21]. During the “wet” winding of the fibres, the fibres were pre-tensioned. This, in turn, caused a liquid resin to flow out from under them, thus reducing the value of this tension. Corden et al. [22] presented the impact of resin shrinkage during the composite cylinder moulding process on the value of radial stresses along the wall thickness. The mechanical properties of the fibre and resin in the laminate were measured and used to determine interlaminar mechanisms which cause high stress and cracking. Thick-walled tubular sections of various laminate structures were used for the analysis and this investigation revealed that if the preform does not limit the shape of the manufactured element, then no internal stress is generated, and thus the matrix cracks do not occur. For moulded parts, the resin shrinkage was inhibited by the stiffer fibres and therefore strains and cracks arise. The study [23] described the measurement and analysis of interlaminar deformations and on the outer surface in a multilayer filament-wound composite pipe. Carbon fibres and glass fibre-reinforced epoxy resin were used for the winding. During the fabrication process, strain gauges and fibre optic sensors were placed between successive layers of the composite. Then, strength tests were performed to measure the circumferential strain on the outer surfaces of individual layers of the structure.

In [24], pipes made of glass fibre-reinforced material (GFRP) were designed with four different winding angles [±45/±45/±45], [±55/±55/±55], [±63/±63/±63] and [±63/±45/±55]. Each tube had an internal diameter of 110 mm, a wall thickness of 3.8 mm and was 450 mm long. The structures were exposed to internal pressure. The paper [25], discusses carbon-fibre-reinforced polymer (CRFP) composites for repairs of damaged steel pipes. The subject of the study involved the effects of defect dimensions and mechanical properties of the repair layer on the burst performances of the repaired pipes. These were done using burst tests and finite element (FE) analysis. In [26], a three-dimensional finite element model was developed for the analysis of stresses in a multilayer pipe subjected to the combination of both the internal pressure and the thermal load, and it incorporated the evaluation of temperature-dependent dependencies. Optimal reinforcement angles were determined for the axially restrained ends (as is a long pipeline) and closed ends (as in a pressure vessel). The influence of uniform and non-uniform heating on failure resulting from stress was assessed. In [27], the ultimate failure of the woven composite pipes was investigated using progressive damage modelling. The composite pipe specimens were made of (E) glass plain weave fabrics according to the ASTM D2290 standard. The hoop strength of these specimens was obtained from the tensile tests. The damage initiation and propagation in the case of a composite pipe were predicted by a numerical multi-scale method.

Based on the analysis of the literary sources, the authors concluded that despite a sizable number of scientific studies in the field of cylindrical composite structure manufacturing and testing, the problem of their broadly understood strength remains a scientifically valid issue. In the majority of the works that studied the state of deformation/stress in composite cylindrical elements, a given winding tension was assumed (of such a value that the fibre would not be “loose” during the winding process), but as a rule, it is not taken into account in the modelling of the stress state. Therefore, a novel element of the research discussed by the authors in this paper in relation to the current state-of-the-art is the introduction of controlled and programmed stretching of the fibre bundles during the modelling and production of filament-wound composite structures.

## 2. Mathematic Model

Based on the state-of-the-art, the process of analytical modelling of a tubular composite structure was carried out, beginning with a single-layer object and concluding with a multi-layer object.

### 2.1. Analysis of the Single-Layer Pipe Fabricated of Anisotropic Material under Internal and External Pressure Loading

We assume that the pipe material satisfies the conditions specified for orthogonal anisotropy, where each element is characterized by three axes of symmetry. The first coincides with the circumferential direction of the pipe, the second with the radial direction, and the third is perpendicular to the others. During hoop-winding, a created material fulfilled the above-mentioned orthotropic conditions.

The problem is considered analogously to the well-known Lamé Problem concerning the stress distribution in a thick-walled cylinder under conditions of plane stress state [4,8,28,29]. In such an arrangement, the plane strain components in the pipe’s cross-section are equal to zero, which in practice means that the pipe is free to deform in an axial direction.

**Figure 1:**
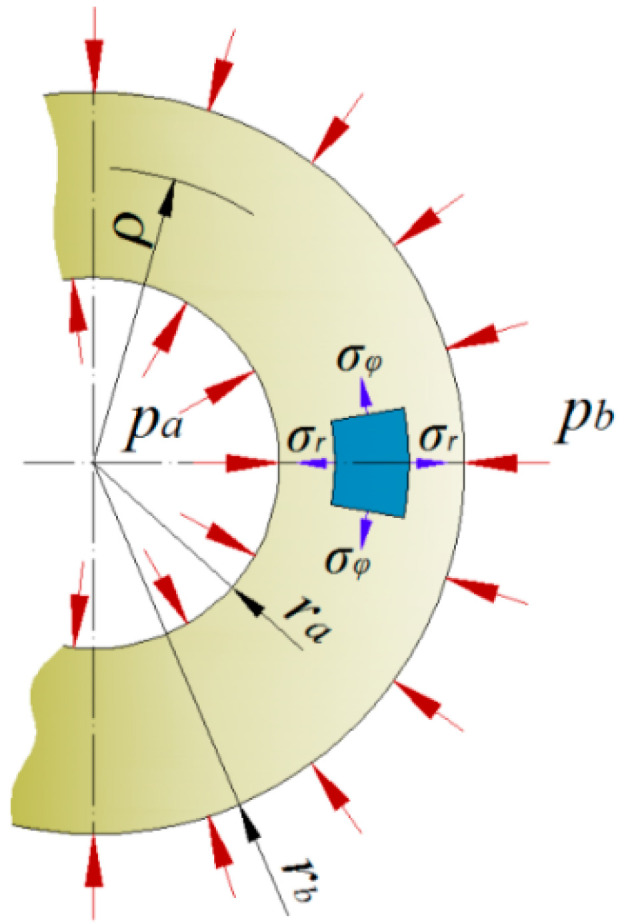
Model of a single layer pipe established for the calculations, where *p_a_*—internal pressure, *p_b_*—external pressure, *r_a_*—internal radius, *r_b_*—external radius and *ρ*—concentric surface radius.

The determination of stress, strain and radial displacement in a layer is possible at any distance ρ from the axis of the pipe when the following values are known (Figure 1):

- material constants: *Ε_r_, E_ϕ_, ν_rϕ_,*
- the pressure exerted on the external surface (*p_b_*) and pressure exerted on the internal surface (*p_a_*),
- internal radius (*r_a_*) and external radius (*r_b_*).

The relationship between the stresses and the strains in a plane stress state is described by the following constitutive equations:

(1) $$\varepsilon_{r}=\frac{\sigma_{r}}{E_{r}}-\frac{\nu_{\phi r}\sigma_{\phi }}{E_{\phi }},\;\varepsilon_{\phi }=\frac{\sigma_{\phi }}{E_{\phi }}-\frac{\nu_{r\phi }\sigma_{r}}{E_{r}},\;\gamma_{r\phi }=\frac{\tau_{r\phi }}{G_{r\phi }}.$$

In the described relationships, the *ϕ* index corresponds to the circumferential direction, while the *r* index denotes the radial direction. In the problem considered circular symmetry is assumed, which means that non-dilatational strain *γ_rϕ_ =* 0, therefore *τ_rϕ_ =* 0.

As far as Poisson’s ratio *υ* is concerned, the first letter in the subscript corresponds to the loading direction, whereas the second one corresponds to the strain direction. The indices at the modulus of elasticity *E* indicate the direction of the strain.

After determining the integration constants for the equilibrium differential equation for the circular-symmetric problem and having made appropriate reductions, the formulas describing the components of the hoop and radial stress depending on the radius *ρ*, where: *ρ* *ϵ* (*r_a_, r_b_*) shall assume the following form [30]:

(2) $$\sigma_{r}\left(\rho \right)=\frac{\rho^{-k-1}\left(p_{b}r_{b}^{k+1}\left(\rho^{2k}-r_{a}^{2k}\right)-p_{a}r_{a}^{k+1}\left(\rho^{2k}-r_{b}^{2k}\right)\right)}{r_{a}^{2k}-r_{b}^{2k}},\;\sigma_{\phi }\left(\rho \right)=\frac{k\rho^{-k-1}\left(p_{b}r_{b}^{k+1}\left(r_{a}^{2k}+\rho^{2k}\right)-p_{a}r_{a}^{k+1}\left(r_{b}^{2k}+\rho^{2k}\right)\right)}{r_{a}^{2k}-r_{b}^{2k}}.$$

where anisotropy coefficient $k=\sqrt{\frac{E_{\phi }}{E_{r}}}$.

For plane stress state *σ_z_ = 0*.

Radial and hoop strains can be determined using constitutive Equation (1):

(3) $$\varepsilon_{r}\left(\rho \right)=\frac{1}{E_{r}}\sigma_{r}\left(\rho \right)-\frac{v_{\phi r}}{E_{r}}\sigma_{\phi }\left(\rho \right)=\frac{\rho^{-k-1}\left(p_{b}r_{b}^{k+1}\left(\rho^{2k}-r_{a}^{2k}\right)-p_{a}r_{a}^{k+1}\left(\rho^{2k}-r_{b}^{2k}\right)\right)}{E_{r}(r_{a}^{2k}-r_{b}^{2k})}-\;-\frac{k\rho^{-k-1}v_{\phi r}\left(p_{b}r_{b}^{k+1}\left(r_{a}^{2k}+\rho^{2k}\right)-p_{a}r_{a}^{k+1}\left(r_{b}^{2k}+\rho^{2k}\right)\right)}{E_{\phi }(r_{a}^{2k}-r_{b}^{2k})},\;\varepsilon_{\phi }\left(\rho \right)=\frac{1}{E_{\phi }}\sigma_{\phi }\left(\rho \right)-\frac{v_{r\phi }}{E_{r}}\sigma_{r}\left(\rho \right)=\frac{k\rho^{-k-1}\left(p_{b}r_{b}^{k+1}\left(r_{a}^{2k}+\rho^{2k}\right)-p_{a}r_{a}^{k+1}\left(r_{b}^{2k}+\rho^{2k}\right)\right)}{E_{\phi }(r_{a}^{2k}-r_{b}^{2k})}-\;-\frac{\rho^{-k-1}v_{r\phi }\left(p_{b}r_{b}^{k+1}\left(\rho^{2k}-r_{a}^{2k}\right)-p_{a}r_{a}^{k+1}\left(\rho^{2k}-r_{b}^{2k}\right)\right)}{E_{r}(r_{a}^{2k}-r_{b}^{2k})}.$$

The radial displacement of any point of the pipe, located at a distance ρ from its axis, can be determined from the Equation (4).

(4) $$\varepsilon_{\phi }=\frac{u}{\rho }.$$

Based on (4), the following can be concluded:

(5) $$u\left(\rho \right)={\rho \varepsilon }_{\phi }\left(\rho \right)=\frac{1}{E_{\phi }E_{r}\left(r_{a}^{2k}-r_{b}^{2k}\right)}\rho^{-k}\left\{p_{b}r_{b}^{k+1}\left[kE_{r}\left(r_{a}^{2k}+\rho^{2k}\right)-E_{\phi }\nu_{r\phi }\left(\rho^{2k}-r_{a}^{2k}\right)\right]\;-p_{a}r_{a}^{k+1}\left[kE_{r}\left(r_{b}^{2k}+\rho^{2k}\right)-E_{\phi }\nu_{r\phi }\left(\rho^{2k}-r_{b}^{2k}\right)\right]\right\}$$

### 2.2. Analysis of the Multi-Layer Pipe Fabricated from Materials of Different Properties

Extending this model to a multi-layer system consisting of layers made of materials with different properties requires the introduction of appropriate parameter indexing and certain assumptions.

**Figure 2:**
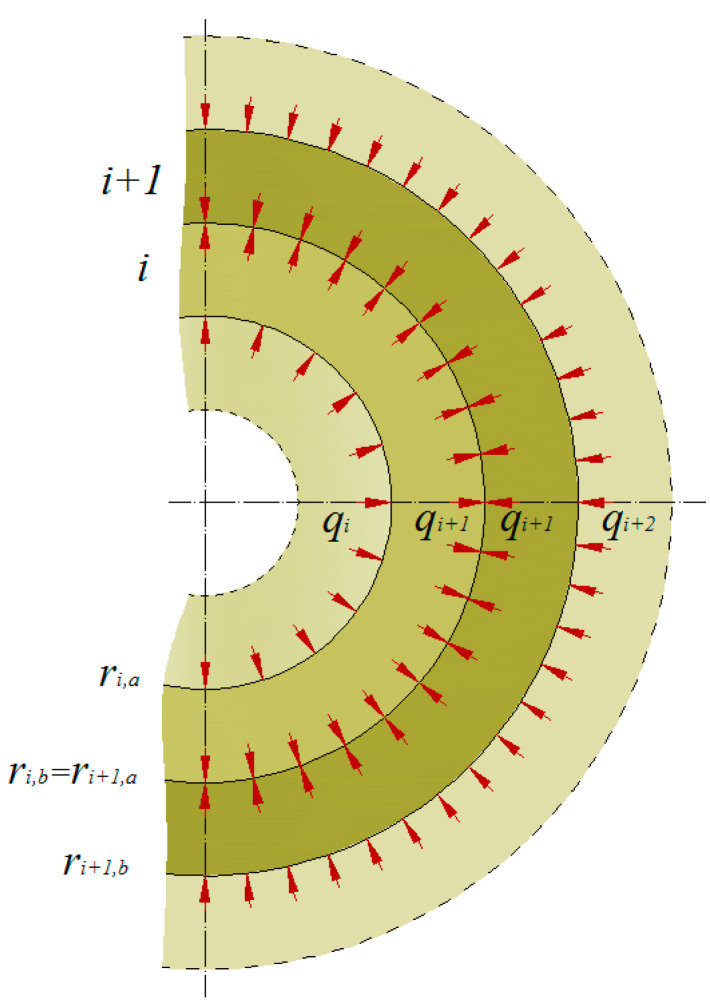
Model of a multi-layer pipe established for the calculations, where *r*—radius, *i*—layer, *q*—inter-layer pressure.

For the *i*-*th* layer (Figure 2), the following dependencies are correct:

(6) $$\sigma_{\phi }^{\left(i\right)}\left(\rho \right)=\sigma_{\phi }\left(E_{i,r},E_{i,\phi },\nu_{i,r\phi },r_{i,a},r_{i,b},p_{i,a},p_{i,b}\right),\;\sigma_{r}^{\left(i\right)}\left(\rho \right)=\sigma_{r}\left(E_{i,r},E_{i,\phi },\nu_{i,r\phi },r_{i,a},r_{i,b},p_{i,a},p_{i,b}\right),\;\varepsilon_{\phi }^{\left(i\right)}\left(\rho \right)=\sigma_{\phi }\left(E_{i,r},E_{i,\phi },\nu_{i,r\phi },r_{i,a},r_{i,b},p_{i,a},p_{i,b}\right),\;\varepsilon_{r}^{\left(i\right)}\left(\rho \right)=\sigma_{r}\left(E_{i,r},E_{i,\phi },\nu_{i,r\phi },r_{i,a},r_{i,b},p_{i,a},p_{i,b}\right),\;u^{\left(i\right)}\left(\rho \right)=u_{r}(E_{i,r},E_{i,\phi },\nu_{i,r\phi },r_{i,a},r_{i,b},p_{i,a},p_{i,b}).$$

As subsequent layers are in surface contact with each other, which is why the following equation is true:

(7) $$r_{i,b}=r_{i+1,a}.$$

and:

(8) $$q_{i+1}=p_{i,b}=p_{i+1,a}.$$

As a result of applying load (pressure) to any of the layers, such a layer will deform, which will force pressure on the subsequent layer. Since it is assumed that the contact surfaces of the layers do not delaminate along the radius, radial displacements of the contacting layers must have the same value, i.e.

(9) $$u^{\left(i\right)}\left(\rho =r_{i,b}\right)=u^{\left(i+1\right)}\left(\rho =r_{i+1,a}\right).$$

Equations (5) and (9) can be written as a linear equation:

(10) $$\alpha_{i}q_{i}+\beta_{i}q_{i+1}+\gamma_{i}q_{i+2}=0,\;i=(1,\ldots ,n-1),$$

where the coefficients:

(11) $$\alpha_{i}=\frac{2k_{i}r_{i,a}^{k_{i}+1}r_{i,b}^{k_{i}}}{\left(r_{i,a}^{2k_{i}}-r_{i,b}^{2k_{i}}\right)E_{i,\phi }},\;\beta_{i}=-\frac{k_{i+1}r_{i+1,a}(r_{i+1,a}^{2k_{i+1}}+r_{i+1,b}^{2k_{i+1}})}{\left(r_{i+1,a}^{2k_{i+1}}-r_{i+1,b}^{2k_{i+1}}\right)E_{i+1,\phi }}-\;-\frac{\left(k_{i}r_{i,b}\left(r_{i,a}^{2k_{i}}+r_{i,b}^{2k_{i}}\right)E_{i,r}E_{i+1,r}+\left(r_{i,a}^{2k_{i}}-r_{i,b}^{2k_{i}}\right)E_{i,\phi }\left(r_{i,b}E_{i+1,r}\nu_{i,r\phi }-r_{i+1,a}E_{i,r}\nu_{i+1,r\phi }\right)\right)}{\left(\left(r_{i,a}^{2k_{i}}-r_{i,b}^{2k_{i}}\right)E_{i,r}E_{i,\phi }E_{i+1,r}\right)},\;\gamma_{i}=\frac{2k_{i+1}r_{i+1,a}^{k_{i+1}}r_{i+1,b}^{k_{i+1}+1}}{\left(r_{i+1,a}^{2k_{i+1}}-r_{i+1,b}^{2k_{i+1}}\right)E_{i+1,\phi }}.$$

*q_1_* and *q_n_*_+1_ values are known and equal: *q*_1_ *= p_0_, q_n_*_+1_ *=* 0 (no pressure on the outer surface of the pipe is assumed). Thus, a nonhomogeneous system of simultaneous equations is obtained.

### 2.3. Analysis of the Multi-Layer Pipe Fabricated from Materials of Different Properties and Loaded with Internal Pressure

**Figure 3:**
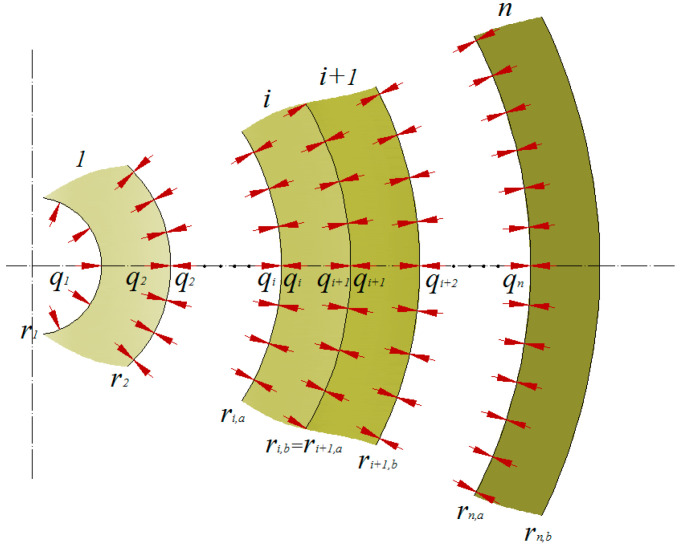
Model of a multi-layer pipe under internal pressure assumed for the calculations, where *r*—radius, *i*—layer, *q*—inter-layer pressure.

The multilayer structure loaded with internal pressure is shown in the diagram (Figure 3). The pipe consists of *n* layers, i.e., the number of contact surfaces between the layers is *n*–1. The number of linearly independent Equation (10) indexed from 1 to *n*–1 is the same.

Model of a multilayer pipe loaded with internal pressure used for calculations.

The *α_i_*, *β_i_* and *γ_i_* coefficients are uniquely defined by the coefficients describing the material properties and the geometry of the layers. Since the pipe is loaded with internal pressure and there is no pressure acting on the external surface, we obtain two additional equations (boundary conditions):

(12) $$\begin{array}{c} q_{1}=p_{1,a}=p_{0},\\ q_{n+1}=p_{n,b}=0. \end{array}$$

The analysed system of equations consists of *q_1_* to *q_n_*_+1_ unknowns and *n* + 1. The calculated vector of interlayer forces, the properties of the materials used, and the radii of the layers introduced to the system of Equation (6) allow for the calculation of the stresses, strains and displacements of any point belonging to the *i-th* layer located at a distance of *ρ* from the pipe axis.

### 2.4. Analysis of the Multi-Layer Pipe Fabricated from Materials of Different Properties and Loaded with Interlayer Pressure

**Figure 4:**
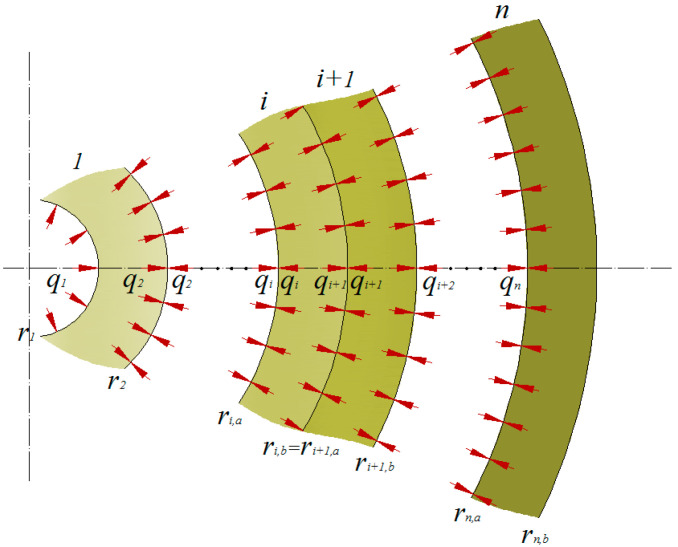
Model of a multi-layer pipe loaded with interlayer pressure assumed for the calculations, where *r*—radius, *i*—layer, *q*—inter-layer pressure.

To create pressure between the layers, the winding of the fibres under tension is proposed. Wrapping the *j*-*th* layer of prestressed material exerts pressure on the previously wound layers, indexed from 1 to *j*–1. This situation is shown in the diagram (Figure 4).

In this case, as before, we assume that the intermediate pressures exerted on the contact surfaces are equal in value:

(13) $$q_{i+1,j}=p_{i+1,j,a}=p_{i,j,b}.$$

In the above equation, the j subscript informs us that the intermediate pressure *q_i_*_+1*, j*_ was generated as the result of wrapping the layer designated with the number *j* and the condition *i* < *j* is apparent.

The pressure of the wrapped layer acting on the previous layers *q_j, j_* can be calculated from the problem of a thin-walled pipe, which is a simplification of the thick-walled pipe problem:

(14) $$q_{j,j}=\lambda_{j}\frac{g_{j}}{r_{j,a}}.$$

where *g_j_* is the thickness of the layer with index *j*, *λ_j_* is the stress resulting from winding tension (filament tension during winding). The system of equations describing the equilibrium of stresses in the layers indexed from 1 to *j*–1 is described by *j*–2 equations:

(15) $$\alpha_{i}q_{i,j}+\beta_{i}q_{i+1,j}+\gamma_{i}q_{i+2,j}=0.$$

Additionally, as shown in Figure 4, we assume the boundary conditions *q*_1, *j*_ = 0, *q_j+_*_1*, j*_ *= 0* and the condition (14). Thus, we shall obtain a system of equations which produces an unambiguous solution. As before, the calculated vector of interlayer forces, material properties and the radii of the layers as substituted for the system of Equation (6) allows for the calculation of stresses, strains and displacements resulting from wrapping the additional *j* layer. These will be designated in the following manner: $\sigma_{n,\phi }^{\left(i,j\right)},\sigma_{n,r}^{\left(i,j\right)},\varepsilon_{n,\phi }^{\left(i,j\right)},\varepsilon_{n,r}^{\left(i,j\right)},u_{n}^{\left(i,j\right)}$, where additional superscript *j* and subscript *n* (winding) denote the reason for their formation.

In layers with numbers from 1 to *j*–1, compressive (negative) hoop stress arises, while in the wrapped layer *j*, tensile (positive) hoop stress is generated.

Several layers are to be added to the structure and wrapped under different tensions, and that is why, according to the principle of stress superposition, the stress of the *i-th* layer will be the arithmetic sum of the stresses generated independently of various factors, which can be written by the following equation:

(16) $$\begin{array}{c} \sigma_{n,r}^{\left(i\right)}=\overset{n}{\underset{k=1}{\sum }}\sigma_{n,r}^{\left(i,k\right)},\\ \sigma_{n,\phi }^{\left(i\right)}=\overset{n}{\underset{k=1}{\sum }}\sigma_{n,\phi }^{\left(i,k\right)}. \end{array}$$

### 2.5. Modelling Results

The independently calculated stress components resulting from the fibre tension (pressures between the layers) and the pipe internal pressure load can be written as the sum of:

(17) $$\begin{array}{c} \sigma_{r}^{\left(i\right)}=\sigma_{n,r}^{\left(i\right)}+\sigma_{p,r}^{\left(i\right)},\\ \sigma_{\phi }^{\left(i\right)}=\sigma_{n,\phi }^{\left(i\right)}+\sigma_{p,\phi }^{\left(i\right)}. \end{array}$$

Based on the above relationships, the stress components can be used to calculate the stress within individual layers and to analyse the strength of the entire multilayer structure.

## 3. Double-Layer Ring Strength Testing

**Figure 5:**
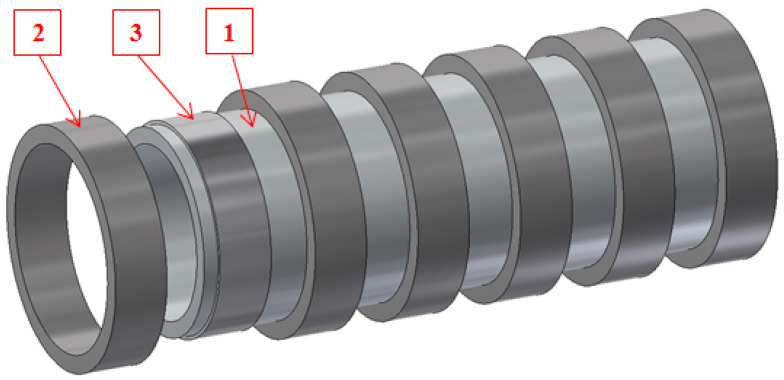
Core prepared for sample winding; 1—core (mandrel), 2—steel ring, 3—steel insert.

In the next stage of work, two-layer samples were manufactured, consisting of an inner steel layer and an outer, epoxy-carbon, layer. The 2-mm-thick steel inserts were placed on the core between the steel rings, which determined the width of the samples, and then carbon fibres were wrapped around them (Figure 5). The steel inserts were used so the stresses arising during winding and resulting from the tensile force would remain at the boundary of the layers and would not transfer to the steel core.

Five samples were manufactured to conduct the tests, and for each sample the value of the tension of the fibres (rovings) was different. The parameters of the samples were shown in Table 1.

The composite layer of the test specimens was made of carbon fibre (TENAX™ UTS 5631 12K (Toho Tenax, Tokio, Japan)) and epoxy resin (Epolam 5015 (Axson, Cergy, France)).

**Figure 6:**
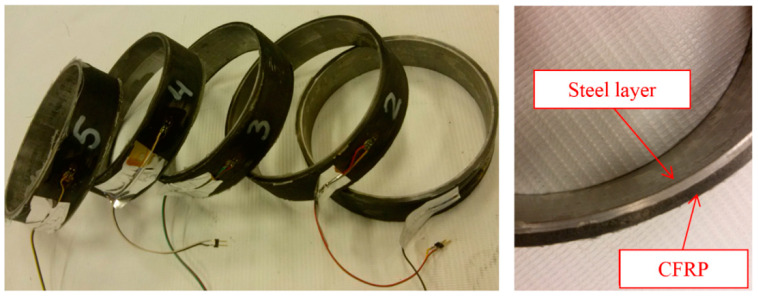
Manufactured samples.

After the samples were manufactured, they were removed from the core and prepared for a tensile test on a testing machine. During removal from the core, the rings exhibited no resistance, which means that the steel layer did not deform under the tensile forces of the fibres during winding. The samples are shown in Figure 6.

Working on the study, the authors also investigated the strength parameters of the manufactured composite structures. For microscopic tests, additional rings (5) were made, wound with the same forces as was used in the samples subjected to strength tests. Afterwards, fragments of the structure were cut out of those rings and the fragments under-went grinding, polishing and washing. Microscopic analysis was performed using an optical microscope and SEM.

The obtained microscopic images of the structures were analysed in terms of two factors:

- the number and distribution of defects in the form of discontinuities in the matrix filling,
- surface distribution and fibre volume ratio within the composite.

The image analysis method was also used to determine the fibre volume ratio, matrix and voids within the analysed structures.

The basic assumption is that the assessment of the fibre distribution in the two-dimensional cross-section is representative of the volume ratio. This method is mainly used to study the distribution of fibres characterised by uniform cross-section, as in the case of the structures investigated in our paper. The method is described in more detail in Composite Materials Handbook [31].

This technique requires the preparation of metallographic specimens of the samples. Then, under a microscope with a magnification of at least 400×, photographs are taken and analysed using specialized image analysis software.

The method aims to distinguish the colour boundary between the fibres and the matrix, therefore in the first stage, the image should be reduced to grayscale. The threshold value of the individual colours can be established by analysing the histogram.

The histogram presents the ratio of grey and black (fibres and resin, respectively). The computer then counts the number of pixels for the corresponding colours, and the ratio of these values to the total number of pixels determines the percentage of each component in the composite.

**Figure 7:**
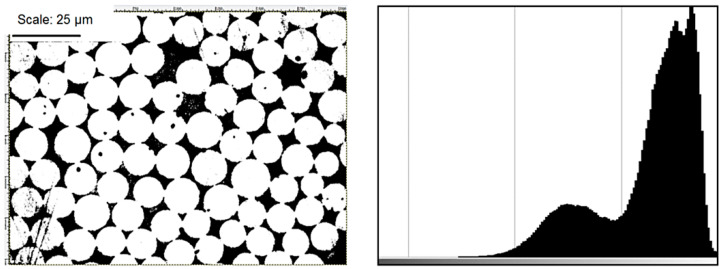
Example of a structure and its histogram.

Figure 7 illustrates an example of a cross-section of the analysed structure with a visible border between individual phases and a histogram.

The prepared surfaces of the samples were studied under the microscope at the magnification of:

- 50×, to determine the distribution of the roving strands and the percentage of voids within the composite,
- 200×, to illustrate the size of the discontinuity of the structure,
- 500×, to determine the distribution of fibres within the matrix and their percentage within the composite.

**Figure 8:**
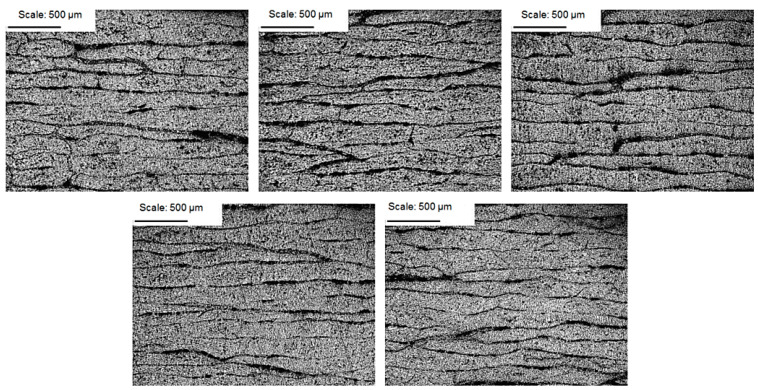
CFRP composite microstructure photomicrographs; magnification of 50× for samples 1–5.

**Figure 9:**
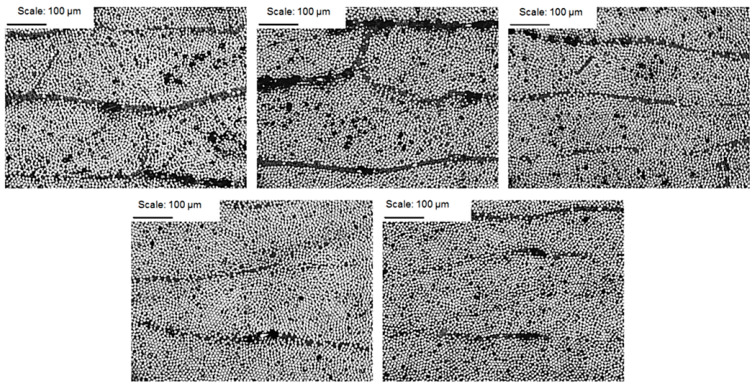
CFRP composite microstructure photomicrographs; magnification of 200× for samples 1–5.

**Figure 10:**
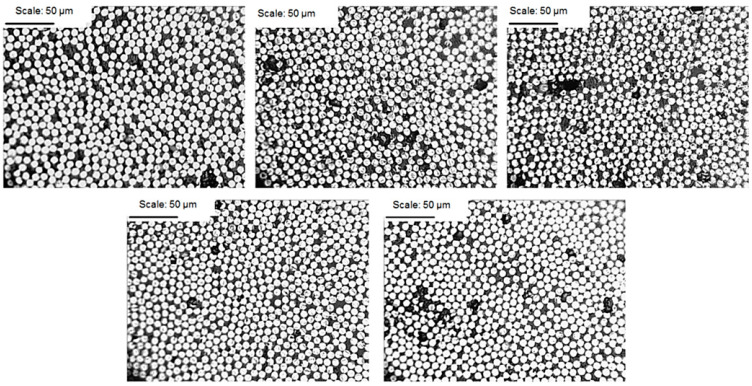
CFRP composite microstructure photomicrographs; magnification of 500× for samples 1–5.

Examples of the cross-sections of the investigated structures for the EW composite are shown in Figure 8, Figure 9 and Figure 10.

**Figure 11:**
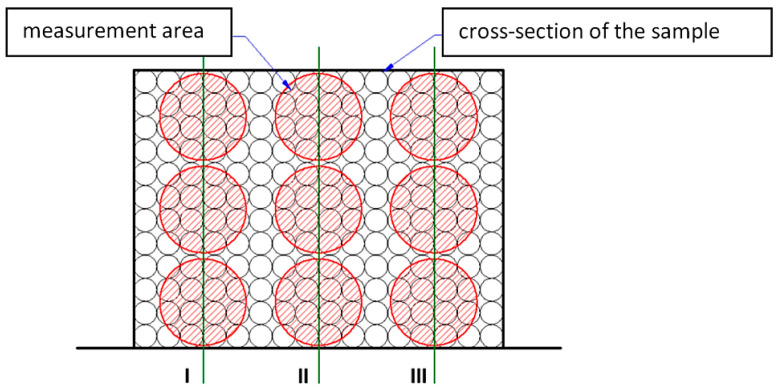
Distribution of the studied nine areas on the sample cross-section.

The volumes of voids and discontinuities in the matrix, as well as the volume of the fibres, were determined by measuring the percentage of surface areas occupied by the respective components of the composite. Each cross-section of individual samples was photographed in three planes parallel to the layers, and three planes perpendicular to the layers in a matrix system (Figure 11).

In the first stage, the ratio of voids and discontinuities within the structure was examined at a magnification of 50×. After determining the void content, the contents of the remaining composite components, i.e., fibres and resin, were analysed at a magnification of 500× (Table 2).

**Figure 12:**
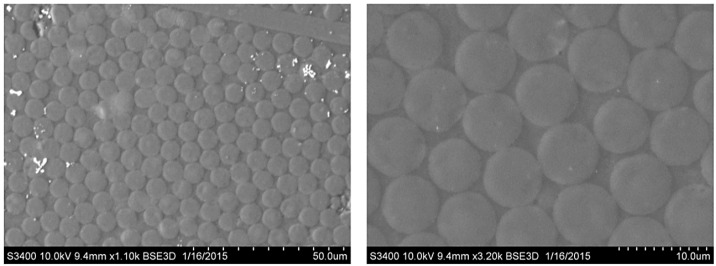
CFRP composite microstructure photomicrographs; magnification of 1100× and 3200× for sample 5.

Additionally, photographs were taken on the SEM/HITACHI S-3400N/2007 scanning electron microscope (Department of Mechanics, Materials and Biomedical Engineering, Faculty of Mechanical Engineering, Wroclaw University of Technology, Wroclaw, Poland), Figure 12.

To determine the effective properties of the composite, the homogenization method based on Eshelby’s inclusion problem was used, expanded to include cyclic boundary conditions. Calculation results are presented in Table 3, whereas the performed process was described in [32,33,34].

While preparing samples for testing, strain gauges were glued onto the samples’ external surfaces to measure the circumferential strains generated as the result of the applied internal pressure. The rings were then successively loaded on the inner surface.

**Figure 13:**
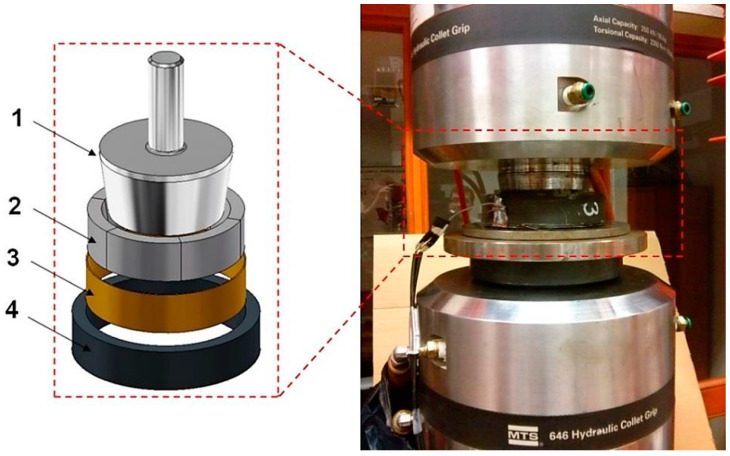
Strength test ring.

The tests were performed on a prepared test stand consisting of a special device mounted in an MTS series 809 hydraulic axial-torsional test system (Department of Mechanics, Materials and Biomedical Engineering, Faculty of Mechanical Engineering, Wroclaw University of Technology), Figure 13.

The testing machine comprises a taper die (1), which is pressed into a split ring (2), placed inside the tested composite ring (4). An insert (3), improving uniformity of internal pressure distribution exerted on the test sample, was placed between the split ring and the composite ring. Due to the mutual contact of these elements, the vertical force exerted by the testing machine head changes its direction to the horizontal component of the force. The punch had a 1:7.15 taper that made it possible to attain a much greater force on the surface of the split ring conical surface. It consists of six identical elements, arranged symmetrically around the circumference of the sample. This makes each element move slightly after loading, resulting in an almost even loading on the inner surface of the studied sample.

**Figure 14:**
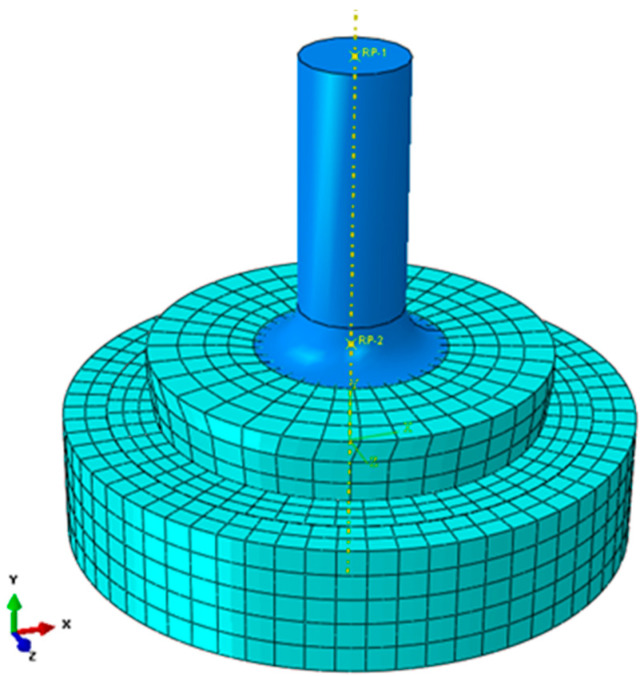
Discrete model of the test rig.

In order to verify the behaviour of individual components during the operation with one another, a model device was fabricated (Figure 14), and then discretized in the Abaqus system and subjected to a preliminary strength analysis (Calculations have been carried out in Wroclaw Centre for Networking and Supercomputing, http://www.wcss.pl, accessed on 14 November 2021).

The following settings were used to create the discrete model:

- Higher-order elements; the geometry of the tested component was not complicated and it was homogeneous (the mesh size was matched to it). The grid was selected based on the previously conducted analyses; the number of elements: 4367 C3D20R elements 20-node hexahedron elements with reduced integration; the number of nodes: 23 129;
- Contacts: tangential behaviour, penalty method, coeff. of friction: 0.1; normal behaviour, “hard contact” pressure overclosure, penalty method;
- Linear models of the material (elastic range);
- Analysis: static general (nonlinear static analysis).

**Figure 15:**
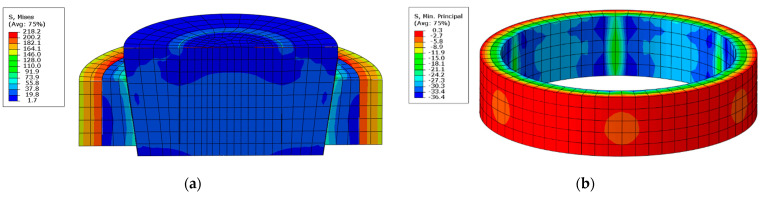
Results of the FEM analysis of the interaction of the split ring with the sample; reduced stresses (**a**), radial stresses: visible contact areas (**b**).

Afterwards, an analysis was performed and it revealed that the elements of the split ring cause significant contact stresses in areas, where their side edges were in contact with contact the sample surface (Figure 15).

To avoid such a situation, an insert was placed between the split ring and the test sample. The 1 mm-thick inserts were made in three versions, each of different materials: including polymer, copper, and elastomer.

**Figure 16:**
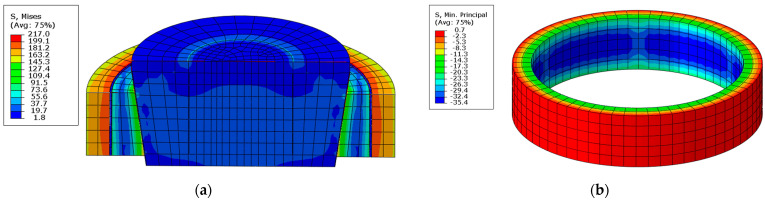
Results of the FEM analysis of the interaction of the split ring with the sample; reduced stresses (**a**), radial stresses: more favourable contact zone areas (**b**).

FEM analysis was performed once again to corroborate the validity of the assumptions. The results (for the polymer insert) are presented in Figure 16.

After the analysis, it was found that the use of the insert significantly improved the homogeneity of the pressure distribution.

Based on the tests and accounting for the different types of inserts, it appears that the most reproducible results were obtained for the copper insert. Copper is a soft material (when compared to steel) and the nature of its deformation is linear. The remaining inserts cause a non-linear ring deformation. Another advantage of the insert is that it prevents deformation on the outer surface of the cone and the inner surface of the split ring caused by their mutual contact. This extends the service life of the device. The insert is a replaceable element, it can be replaced after each test; moreover, the insert manufacturing costs are low.

Strain gauges were attached to external surfaces of the samples (Figure 6) to measure the circumferential strain of the testes composite samples following loading.

**Figure 17:**
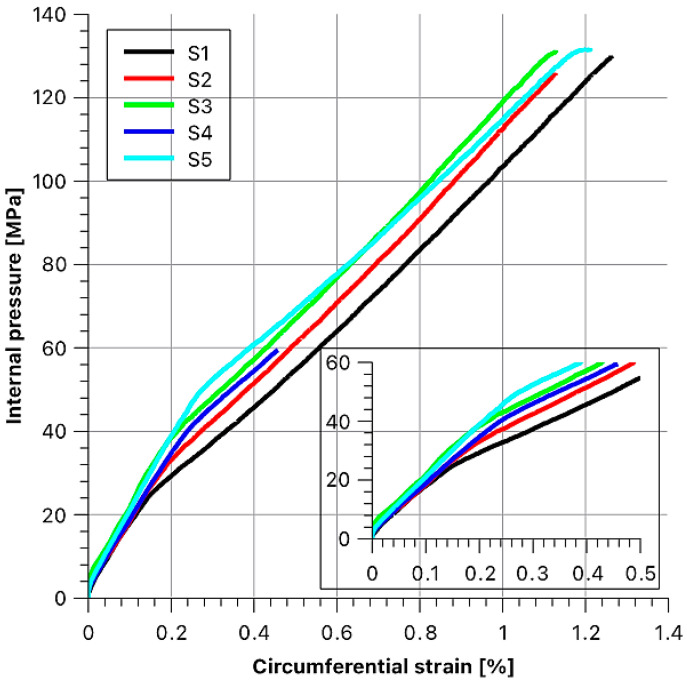
Sample strain results as a function of internal pressure.

Based on the data obtained from the measurements, the hoop strain curve (Figure 17) was drawn as a function of the internal pressure. The maximum possible value of the pressure acting on the internal surfaces of the samples under tension loading was 140 MPa.

Based on the graph presented in Figure 17, two distinct slopes of the function can be seen, and these indicate that there are two sample stiffness moduli. The first range (part of the line) corresponds to the transfer of stresses through the steel layer, and following its plasticization, the load is transferred mainly via the composite layer. The breakdown of the function behaviour is also crucial in this case, as for subsequent samples, the bend in the curve shifts upward, towards higher loads. What follows is that with the increase of the tension of the fibres during winding, the values of stress between the layers were rising. At this point, what must be explained is the course of the function for the “S4” sample S4, which is different from the others.

It was due to damage to the strain gauge with a strain of about 0.45%. During load-ing, the fibre strand broke to which the strain gauge was attached. The “S4” sample, like all the other samples, withstood a strain of almost 1.2%.

The values of the pressure loading the rings and the corresponding strain are summarized in Table 4. These values were determined by drawing lines tangent to the obtained functions and then noting the point at their intersection. The intersection point for sample S1 was set as the “0” point.

**Figure 18:**
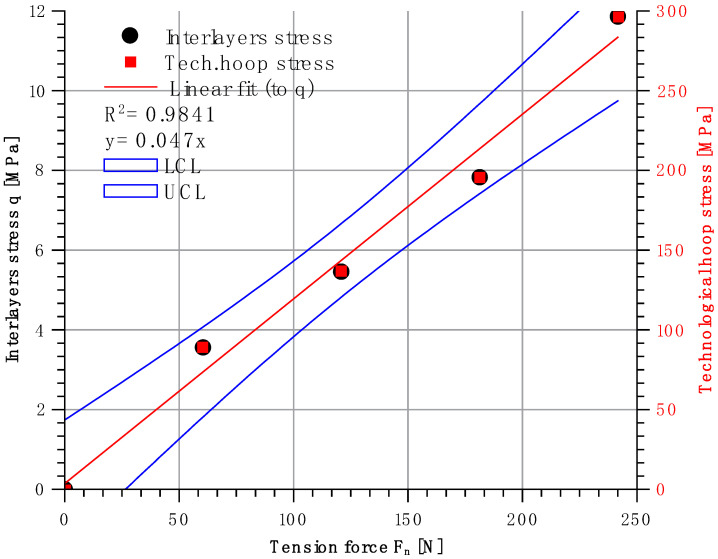
Dependence of interlayer pressure in function of fibre tension force, where LCL—lower control limit, UCL—upper control limit.

Based on the results of the measurements shown in Table 4, the relationship between the interlayer pressure (stress) between the steel insert and the CFRP composite as a function of the fibre strands tension was drawn (Figure 18). Interlaminar pressure values were determined using the developed calculation model, as described in Chapter 1.

A trend line was drawn for the selected measurement points and equation *q_n_ = f(F_n_).* was determined.

Based on the data obtained, it can be concluded that the scatter of the results is small. The coefficient was marked with the letter *ψ*. When calculating statistical error, its value was determined at the level:

(18)  ψ=0.047 ± 0.003 [MPa/N].

Thus, an equation can be written to define the interlayer (interlaminar) radial stresses using the fibre tension force:

(19) $$q_{n}=\psi \cdot F_{n}.$$

Knowing the radial stresses, hoop stresses in the given layer can be determined through this layer’s geometry (axial stress in the fibre strain during winding):

(20) $$\lambda_{n}=\xi \cdot F_{n},$$

where:

(21) $$\xi =\psi \cdot \frac{r_{2,a}}{g_{2}}.$$

The subsequent part of the experiment involved the determination of theoretical behaviours of the functions based on the graph presented in Figure 17. To this end, it was also necessary to define the model of the steel insert material. The insert material “operated” in the elastic region in the first phase of the strain and the plastic region in the second strain phase.

**Figure 19:**
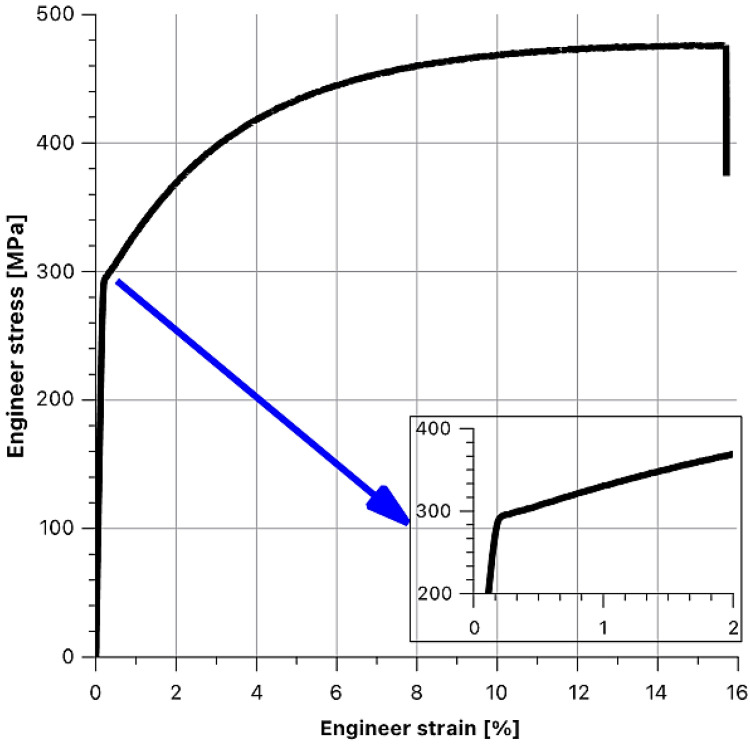
Steel insert material tension test.

To verify this assumption, a strength test of the insert material was performed (Figure 19).

On the basis of the measured values, a bilinear elasto-plastic model was adopted, and the longitudinal modulus of elasticity was determined at the level of E = 204 GPa, E′ = 5.5 GPa. The E′ modulus was determined in the deformation range from 0.2% to 2%, as composite samples were tested in a similar range.

**Figure 20:**
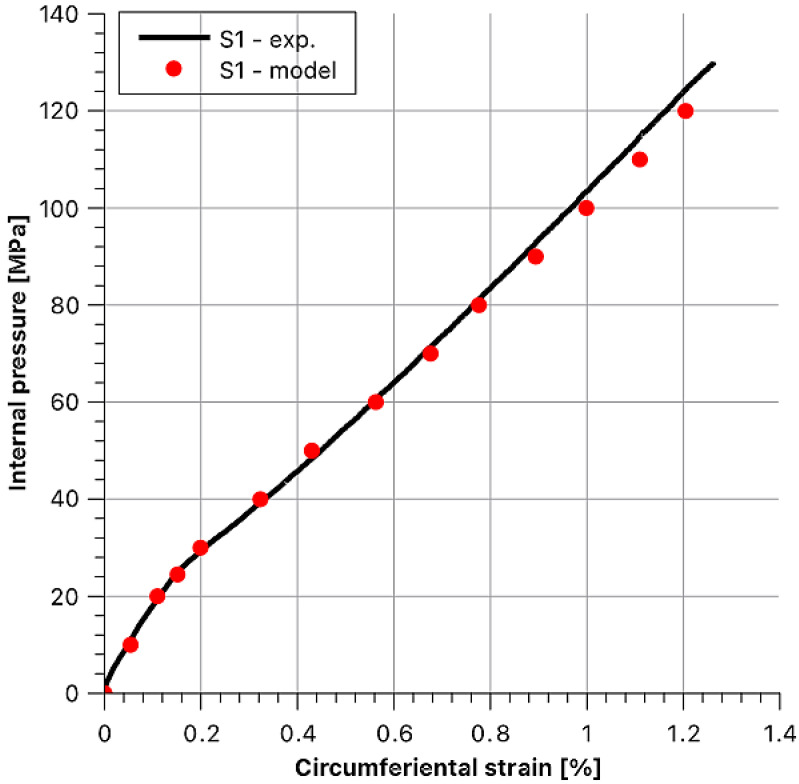
Stress and strain results as a function of internal pressure for sample S1.

**Figure 21:**
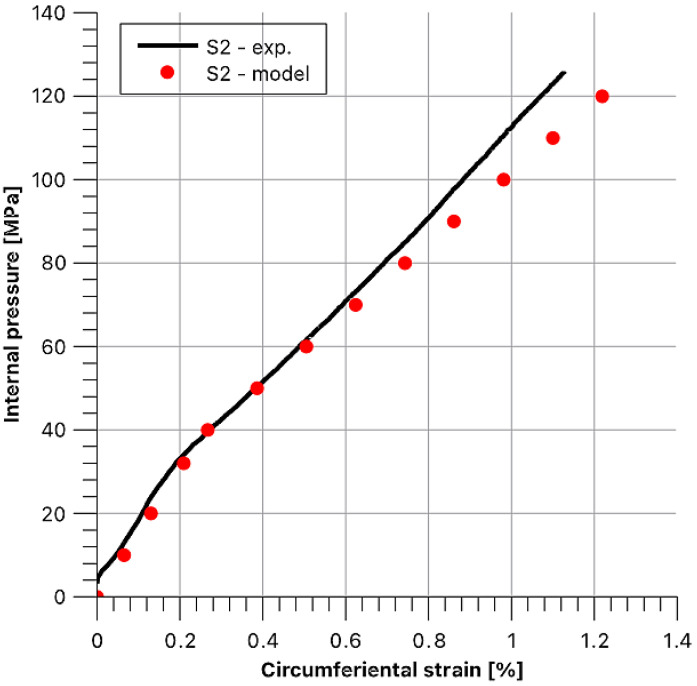
Stress and strain results as a function of internal pressure for sample S2.

**Figure 22:**
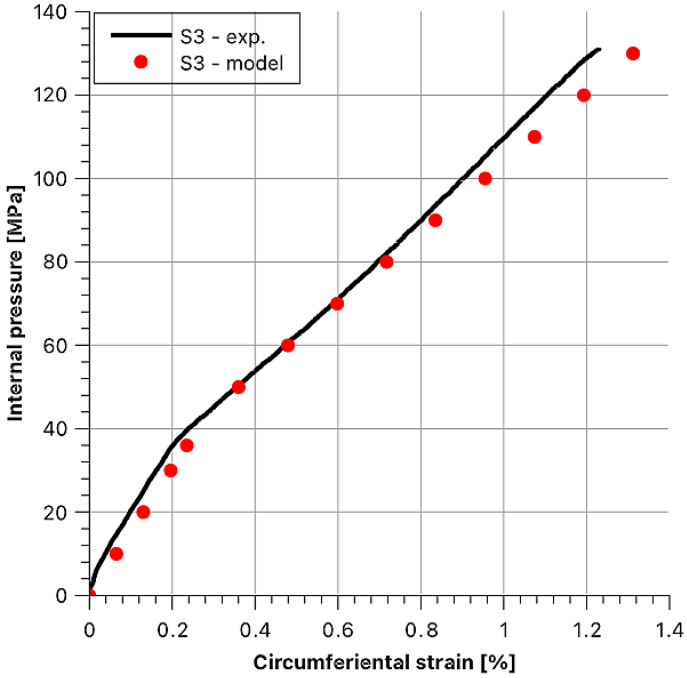
Stress and strain results as a function of internal pressure for sample S3.

**Figure 23:**
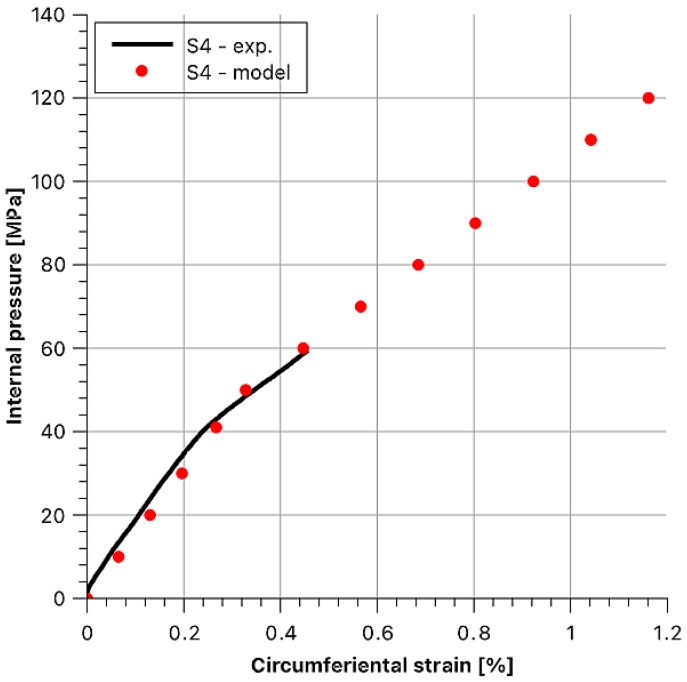
Stress and strain results as a function of internal pressure for sample S4.

**Figure 24:**
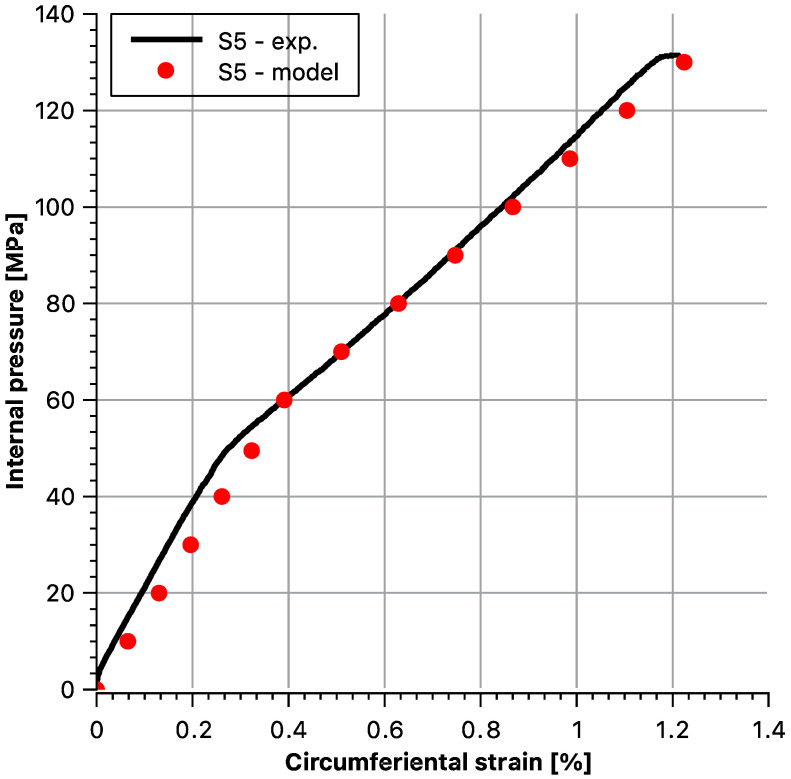
Stress and strain results as a function of internal pressure for sample S5.

Then, theoretical behaviours of the hoop stress/hoop strain function at the outer surface of the sample were determined as dependent on internal pressure. Afterwards, they were compared with the experimental values. To maintain the legibility of the graphs, data for each sample are presented in separate figures (Figure 20, Figure 21, Figure 22, Figure 23 and Figure 24).

## 4. Discussion

In tubular (cylindrical) elements loaded with internal pressure, there are two main stress directions: the circumferential (hoop), and the radial one. In terms of material strength, hoop stresses are the most dangerous. The greatest concentration of hoop stress occurs on the inner surface of the pipe and decreases with increasing wall thickness. To eliminate this disadvantageous distribution, the authors proposed to design the wall as a multilayer structure with appropriate boundary conditions between the layers being introduced simultaneously. This allowed for the design of the material structure characterised by uniform strength along the radius, which enabled the reduction of the weight of the composite and thus the cost of the entire product.

The analytical and experimental tests were aimed at checking if we could obtain the distribution of hoop stress along the radius in the composite multilayer pipe (loaded with internal pressure), which is homogenous as possible, by winding the fibre bundles with appropriate winding tension and/or using materials with differing Young’s modulus. We began our work by modelling the analysed structure based on the classic Lamé Problem. Due to the anisotropic properties of the material, the coefficient of anisotropy k was introduced to describe the modelled structures, as it binds the material’s directional moduli of rigidity.

**Figure 25:**
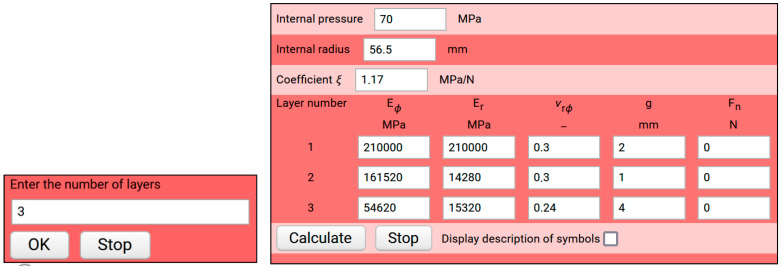
Dialogue window of the structure data.

Based on the tests we conducted, we could determine the relationship between the fibre bundle tension force during winding and the strength of the rings. The graphs of functions (Figure 17) are bilinear. We noticed that along with increasing fibre tension, the inflexion point of the graph shifts towards the higher pressure, causing the change of Young’s modulus. For the sample wound with the force of 18 N, this value is about 25 MPa, while for the sample wound with the force of 260 N it is about 50 MPa. This gives rise to the conclusion that it is possible to control the production process in such a way that a given structure would operate properly under a higher load while maintaining appropriate rigidity. To illustrate this more clearly, we simulated the optimal tension force in individual composite layers by implementing the developed mathematical model to the Wolfram Mathematica system. After defining the number of layers and confirming it, another dialogue window appears, in which you should define the properties of the materials used for individual layers, the thickness of the layers and the winding tension for the fibre strands if the layer consists of a filament-wound composite (Figure 25). Moreover, the value of the ξ coefficient should be given, as it links the hoop stresses in the given layer with the winding tension of the roving strand.

**Figure 26:**
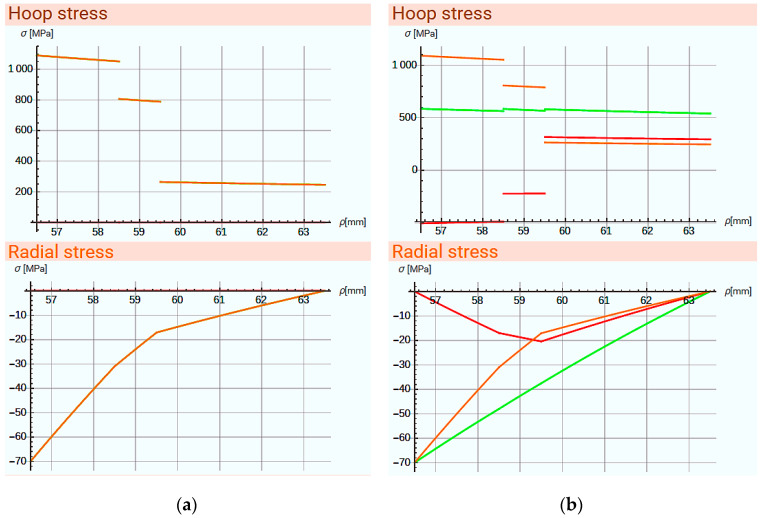
Dialogue window of calculation results. The colour of the graphs represents: **―** stresses due to internal pressure, **―** stresses resulting from the introduction of fibre tension, **―** stresses after optimization.

After the structure data are entered and confirmed, calculations are performed, and another dialogue box appears containing:

- information on the defined layers,
- calculated values and a diagram of hoop stresses (Figure 26),
- calculated values and a diagram of radial stresses (Figure 26),
- calculated values and a diagram of circumferential strain and radial displacements.

Figure 26a shows the uneven distribution of hoop stresses, which are the most interesting values from the point of view of ring strength. To perform the optimization, the maximum tension force at one’s disposal needs to be defined. Then the program selects the values, so as to minimize the maximum hoop stresses. It was assumed that the standard deviation of the stress values tends towards the minimum (optimization criterion). The results and the calculated values are shown in Figure 26b.

**Figure 27:**
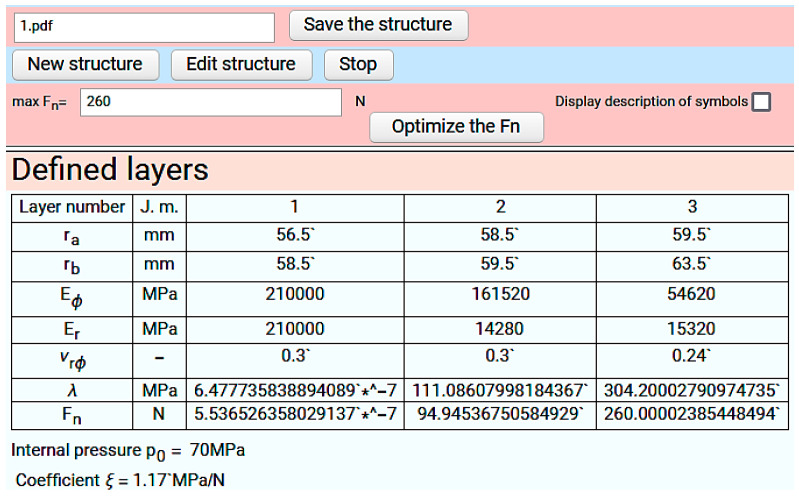
Calculation results of fibre tension force.

The fibre tension force F_n_ that should be used during winding appears in the last row of the table in the “defined layers” window (Figure 27).

Another analysis concerns the strength properties. Based on the microscopic examinations, we could state that fibre volume fraction was at the level of 67%. Therefore, thanks to the high content of fibres and their arrangement in a single direction within the matrix, we obtained high strength parameters of the structures produced. Based on the diagram presented in Figure 17, we were able to conclude that the 4.5-mm-thick composite layers are able to transfer a large load of 130 MPa.

## 5. Conclusions

Our studies included developing a computational model dedicated to cylindrical single-layer and multi-layer composite structures, manufacturing various types of composite structures and subjecting them to a static strength analysis. Based on the modelling and our research, we can state that:

(1) The use of the model to calculate cylindrical composite elements made it possible to define and select appropriate structures in terms of strength;
(2) The accuracy of the assumptions and the model were documented by the high convergence of theoretical and experimental results;
(3) The rigidity and strength of the structure do not depend on the winding tension impacting the fibres (which is visible in Figure 12, where the slopes of the individual functions differ only slightly); this conclusion was also confirmed by the microstructural analysis that revealed that the packing density of the fibres, and thus the strength properties of the composites, are practically independent of the winding tension;
(4) During the manufacture of the multilayer structures, a very important factor to bear in mind is to ensure appropriate stiffness of the liner (the liner cannot be deformed under the impact of stresses resulting from the tension force in the subsequent layer),
(5) The introduced fibre tension generates inter-layer stresses, which cause the resulting distribution of the hoop stress to be more uniform, analogically to the Lamé Problem.

## Figures and Tables

**Table 1:** Parameters of carbon fibre-reinforced polymer (CFRP).

Sample No.	Roving Tension [N]	Thickness of Steel Layer [mm]	Thickness of CFRP Layer [mm] *
S1	18.1	2.0	2.55
S2	78.5	2.0	2.52
S3	138.9	2.0	2.53
S4	199.4	2.0	2.47
S5	259.8	2.0	2.45

* Average cross-section thickness of the sample measured at four points.

**Table 2:** Summary of microstructure surface measurements of CFRP.

	CFRP	
	Volume Fraction of Fibres V_f_ [%]	Volume Fraction of Voids V_v_ [%]
Average	66.9%	7.2%
Standard deviation	3.3%	2.1%
Volume fraction of resin (100–V_f_–V_v_)	25.9%	
Volume fraction of carbon fibre	66.9%	
Mass fraction of carbon fibre	80.4%	

**Table 3:** Effective elastic properties of the manufactured composites.

Properties	Carbon Fibre-Reinforced Polymer (CFRP)
Longitudinal modulus of elasticity E_1_ [GPa]	161.52
Longitudinal modulus of elasticity E_2_ = E_3_ [GPa]	14.28
Rigidity modulus G_12_ [GPa]	5.22
Rigidity modulus G_23_ [GPa]	5.05
Poisson’s ratio ν_12_ [–]	0.30
Poisson’s ratio ν_23_ [–]	0.41

**Table 4:** List of measured values.

Sample No.	Roving Tension F_n_ [N]	Pressure p_0_ [MPa]	Pressure Increase Δp_0_ [MPa]	Strain [%]	Interlaminar Pressure q_n_ [MPa]
S1	18.1	24.5	0	0.145	0
S2	78.5	32.0	7.5	0.185	3.56
S3	138.9	36.0	11.5	0.200	5.46
S4	199.4	41.0	16.5	0.250	7.83
S5	259.8	49.5	25.0	0.270	11.86

## Abbreviations

$\varepsilon_{\phi }$	circumferential strain,
$\varepsilon_{r}$	radial strain,
$\varepsilon_{\phi }^{\left(i\right)}$	circumferential strain of the i-th layer,
$\varepsilon_{r}^{\left(i\right)}$	radial strain of the i-th layer,
$u^{\left(i\right)}$	radial displacement of i-th layer,
$\sigma_{\phi }$	hoop stress,
$\sigma_{r}$	radial stress,
$\sigma_{z}$	axial stress,
$\sigma_{n,\phi }^{\left(i\right)}$	hoop stress of i-th layer from fibre tension,
$\sigma_{n,r}^{\left(i\right)}$	radial stress of i-th layer from fibre tension,
$\sigma_{p,\phi }^{\left(i\right)}$	hoop stress of i-th layer from internal pressure,
$\sigma_{p,r}^{\left(i\right)}$	radial stress of i-th layer from internal pressure,
*E*	Young modulus,
$E^{\prime }$	strain-hardening ratio, post-yield tangent,
$E_{\phi }^{\left(i\right)}$	Young moduli (circumferential direction for i-th layer),
$E_{r}^{\left(i\right)}$	Young moduli (radial direction for i-th layer),
$\nu_{r\phi }^{\left(i\right)}$	Poisson’s ratio that corresponds to a contraction in hoop direction when an extension is applied in radial direction (for i-th layer),
$\nu_{\phi r}^{\left(i\right)}$	Poisson’s ratio that corresponds to a contraction in radial direction when an extension is applied in hoop direction (for i-th layer),
$\gamma_{r\phi }$	shear strain,
$\tau_{r\phi }$	shear stress,
$G_{r\phi }$	shear modulus,
*u*	radial displacement,
$p_{0}$	internal pressure,
$p_{a}$	internal pressure applied for i-th layer,
$p_{b}$	external pressure applied for i-th layer,
$q_{i}$	interlayer pressure applied for i-th layer,
$r_{a}^{\left(i\right)}$	internal radius of i-th layer,
$r_{b}^{\left(i\right)}$	external radius of i-th layer,
ρ	concentric surface radius,
*g_i_*	thickness of i-th layer,
*k*	coefficient of anisotropy,
$F_{n}$	rowing (fibre) tension force,
*λ*	stress resulting from winding tension,
$V_{f}$	fibre volume fraction,
*ψ*	coefficient connecting fibre force tension with interlayer pressure,
*ξ*	coefficient connecting fibre force tension with hoop stress.
